# Simultaneous 3D quantitative magnetization transfer imaging and susceptibility mapping

**DOI:** 10.1002/mrm.30493

**Published:** 2025-03-17

**Authors:** Albert Jang, Kwok‐Shing Chan, Azma Mareyam, Jason Stockmann, Susie Yi Huang, Nian Wang, Hyungseok Jang, Hong‐Hsi Lee, Fang Liu

**Affiliations:** ^1^ Athinoula A. Martinos Center for Biomedical Imaging Massachusetts General Hospital Charlestown Massachusetts USA; ^2^ Radiology Harvard Medical School Boston Massachusetts USA; ^3^ Biomedical Engineering University of Texas Southwestern Medical Center Dallas Texas USA; ^4^ Radiology University of California, Davis Davis California USA

**Keywords:** magnetization transfer, quantitative imaging, quantitative susceptibility mapping, tissue modeling

## Abstract

**Purpose:**

Introduce a unified acquisition and modeling strategy to simultaneously quantify magnetization transfer (MT), tissue susceptibility (χ) and $T_{2}^{*}$.

**Theory and Methods:**

Magnetization transfer is induced through the application of off‐resonance irradiation between excitation and acquisition of an RF‐spoiled gradient‐echo scheme, where free pool spin–lattice relaxation ($T_{1}^{F}$), macromolecular proton fraction (f) and magnetization exchange rate ($k_{F}$) were calculated by modeling the magnitude of the MR signal using a binary spin‐bath MT model with $B_{1}^{+}$ inhomogeneity correction via Bloch‐Siegert shift. Simultaneously, a multi‐echo acquisition is incorporated into this framework to measure the time evolution of both signal magnitude and phase, which was further modeled for estimating $T_{2}^{*}$ and tissue susceptibility. In this work, we demonstrate the feasibility of this new acquisition and modeling strategy in vivo on the brain tissue.

**Results:**

In vivo brain experiments were conducted on five healthy subjects to validate our method. Utilizing an analytically derived signal model, we simultaneously obtained 3D $T_{1}^{F}$, f, $k_{F}$, χ and $T_{2}^{*}$ maps of the whole brain. Our results from the brain regional analysis show good agreement with those previously reported in the literature, which used separate MT and QSM methods.

**Conclusion:**

A unified acquisition and modeling strategy based on an analytical signal model that fully leverages both the magnitude and phase of the acquired signals was demonstrated and validated for simultaneous MT, susceptibility and $T_{2}^{*}$ quantification that are free from $B_{1}^{+}$ bias.

## 1 INTRODUCTION

Quantitative MRI (qMRI) quantifies MR tissue properties, providing information regarding tissue microstructure and microenvironment. It utilizes tissue models based on spin physics to quantitatively measure a tissue parameter of interest (e.g., tissue relaxation, diffusion, perfusion, susceptibility, macromolecular content, etc.). These parameters have been shown to be sensitive to various neurological conditions,^1^ such as dementia,^2^ stroke,^3^ epilepsy^4^ and multiple sclerosis (MS),^5^ demonstrating the potential for usage as imaging biomarkers to assess tissue degeneration. Compared to conventional qualitative MRI, quantitative methods provide superior sensitivity^6^ that could enable better detection of pathologies. It also provides increased specificity^7^ that could allow better identification of disease subtypes.

Quantitative magnetization transfer (qMT)^8^ imaging methods estimate the parameters of a biophysical model to illustrate the MT phenomena investigating the water MR signal affected by macromolecular content in tissue. Commonly utilized is the binary spin‐bath system, which is composed of an aqueous free water pool and a non‐aqueous restricted proton pool to estimate the macromolecular content and its influence on free water $T_{1}$. This model was validated by Henkelman et al. where they showed excellent agreement between the two‐pool model predictions and phantom experiments using continuous wave irradiation.^9^ Sled and Pike further validated this model both ex vivo and in vivo using pulsed irradiation.^10^ Yarnykh^11^ introduced a pulsed Z‐spectroscopic imaging method which estimates a $T_{1}$ map that is subsequently used as a prior to extract the remaining binary spin‐bath parameters. Mossahebi and colleagues modified this two‐step processing pipeline by performing a global fit of the acquired data concurrently, which corrects for bias estimation of qMT parameters when using the two‐step processing approach.^12^ Selective inversion recovery approaches have also been employed to characterize the dynamic nature of the spin‐bath model. Using biexponential longitudinal relaxation to characterize the observable $T_{1}$ as a weighted sum of two longitudinal relaxation exponentials, these were utilized to extract the macromolecular content and exchange rates of the binary spin‐bath model for a single slice.^13^ Soustelle et al. demonstrated a dual‐offset saturation acquisition strategy and a matched mathematical model to compensate for on‐resonance saturation and dipolar order effects in a pulsed spoiled gradient‐echo qMT framework.^14^

Following the line of pulsed MT acquisition, we have recently introduced a new qMT method^15^ that generates $B_{1}^{+}$ inhomogeneity‐corrected free pool spin–lattice relaxation $\left(T_{1}^{F}\right)$ and binary spin‐bath MT parameters, including macromolecular proton fraction and magnetization exchange rate. Instead of placing the off‐resonance MT‐inducing pulse as a preparation module prior to excitation, as was done in the aforementioned methods, our method uses a different approach by putting the off‐resonance irradiation after excitation and before acquisition. This concurrently generates two independent effects: (1) $B_{1}^{+}$ field dependent Bloch‐Siegert shift^16^ and (2) direct saturation of macromolecules. Using an analytical signal model derived at the steady‐state condition, the magnitude of the signal was utilized to quantitatively examine MT effects, while Bloch‐Siegert shift was simultaneously used to correct for $B_{1}^{+}$ bias.

In this work, we extend this sequence framework and signal modeling strategy to effectively acquire more information by incorporating a multi‐echo acquisition. With no additional scan time in comparison to the original method, this added acquisition allows for extra assessment of the time evolution of the signal magnitude and phase along multiple echoes, which can further be leveraged to model and quantify $T_{2}^{*}$ and tissue susceptibility χ. ^17^ By fully considering both the magnitude and phase of the acquired MR signals, we demonstrate the feasibility of this method in vivo where 3D concurrent $B_{1}^{+}$ inhomogeneity‐corrected MT and susceptibility maps were obtained for the whole brain.

## 2 THEORY

### 2.1 Binary spin‐bath system

**FIGURE 1:**
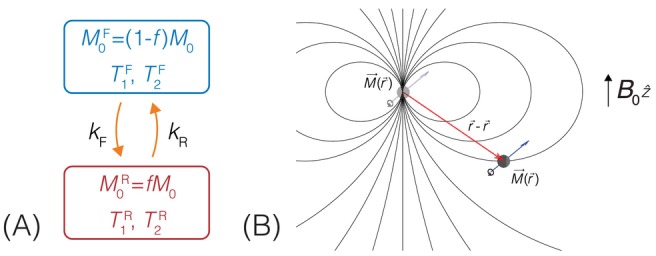
(A) In the presence of macromolecules, the constant exchange of longitudinal magnetization between the “free” pool (blue) and “restricted” pool (red) is modeled by the binary spin‐bath system, where each pool is characterized by a corresponding spin–lattice and spin–spin relaxation constant. $T_{1}^{R}$ = 1 s, $T_{2}^{R}$ = 12 μs was assumed^9^, ^10^, ^21^, ^27^ in this study. (B) Tissue at location $\vec{r^{\prime }}$ generates a magnetic dipole moment in response to externally applied magnetic field $B_{0}\hat{z}$. This induces an additional susceptibility induced field for tissue located at $\vec{r}$.

The binary spin‐bath system is composed of a “free” liquid proton pool F and a “restricted” macromolecule proton pool R (Figure 1A). The exchange of longitudinal magnetization between the two pools is characterized by pseudo‐first‐order rate constants $k_{F}$ and $k_{R}$, where $k_{F}$ is the spin transfer rate from F to R and $k_{R}$ is vice versa. In equilibrium, $k_{F}=kM_{0}^{R}$ and $k_{R}=kM_{0}^{F}$, where k is the fundamental exchange rate constant between pools F and R, and $M_{0}^{F}$ and $M_{0}^{R}$ are the corresponding equilibrium magnetizations of the free pool and restricted pool.

In the rotating frame of reference, the dynamics of the spin magnetization exchange between these two pools can be described by the Bloch‐McConnell equations^18^

(1) ∂MxF(t)∂t=−MxF(t)T2F+∆ωMyF(t)−ImγB1(t)MzF(t)

(2) ∂MyF(t)∂t=−MyF(t)T2F−∆ωMxF(t)+ReγB1(t)MzF(t)

(3) ∂MzF(t)∂t=M0F−MzF(t)T1F−ReγB1(t)MyF(t)+ImγB1(t)MxF(t)−kFMzF(t)+kRMzR(t)

(4) $$\frac{\partial M_{z}^{R}\left(t\right)}{\partial t}=\frac{M_{0}^{R}-M_{z}^{R}\left(t\right)}{T_{1}^{R}}-k_{R}M_{z}^{R}\left(t\right)+k_{F}M_{z}^{F}\left(t\right)-\left\langle W\left(\Delta \right)\right\rangle M_{z}^{R}\left(t\right)$$

where $T_{1}^{F\left[R\right]}$ are the corresponding spin–lattice relaxation times of the free and restricted macromolecule pools, respectively, $T_{2}^{F}$ is the spin–spin relaxation time of the free molecule pool, ∆ω entails any off‐resonance contributions due to static field ($B_{0}\hat{z}$) inhomogeneity and/or variations in the applied RF frequency in units of rad·s^−1^, $B_{1}\left(t\right)$ is the time‐dependent RF pulse in units of T and γ the gyromagnetic ratio in units of rad·s^−1^·T^−1^. ⟨W(Δ)⟩ is the average saturation rate of the macromolecule pool due to irradiation applied at frequency offset Δ, which is proportional to both the macromolecule absorption line shape and the power integral of the applied RF pulse,^19^ given by

(5) $$\left\langle W\left(\Delta \right)\right\rangle =\pi \frac{1}{\tau_{\mathrm{RF}}}\int_{0}^{\tau_{\mathrm{RF}}}{\left|\omega_{1}\left(t\right)\right|}^{2}\mathrm{dtG}\left(\Delta \right)$$

where $\tau_{\mathrm{RF}}$ is the irradiating pulse width, $\left|\omega_{1}\left(t\right)\right|$ is the time‐dependent pulse amplitude (Hz) and G(Δ) is the frequency dependent absorption line shape at an offset Δ with respect to the resonance frequency.

### 2.2 Signal magnitude modeling

**FIGURE 2:**
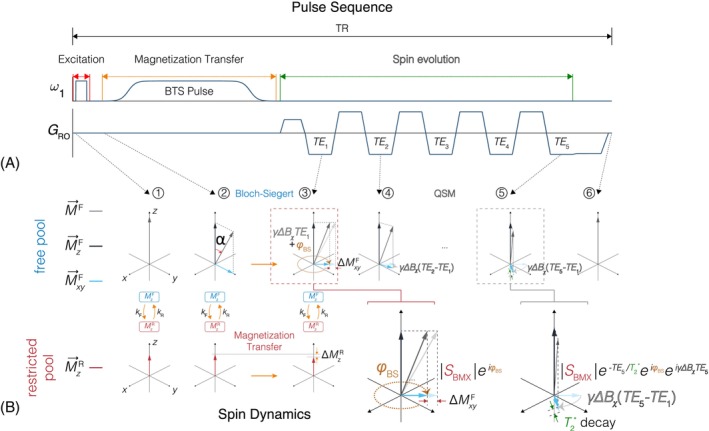
(A) Acquisition uses an RF‐spoiled gradient‐echo scheme composed of a magnetization transfer encoding module (orange) that has an off‐resonance pulse inserted between excitation and acquisition, and a spin evolution module (green) made up of a multi‐echo acquisition scheme. When the pulse complies with the Bloch‐Siegert and magnetization Transfer Simultaneously (BTS) criteria, (B) the transverse magnetization of the free pool acquires a phase proportional to its peak power, while the restricted pool is partially saturated with minimum free pool saturation. This decrease in the restricted pool's longitudinal magnetization due to saturation results in a decrease in the observable transverse magnetization of the free pool with its phase proportional to $B_{1}^{+}$. In the spin evolution module, which is utilized without the off‐resonance pulse applied, magnitude decays and phase evolves as a function of ${\mathrm{TE}}_{n}$. Mono‐exponential decay and tissue susceptibility models are utilized to quantify $T_{2}^{*}$ and tissue susceptibility.

The acquisition sequence is based on a spoiled gradient‐echo scheme composed of a MT encoding module and a spin evolution module (Figure 2A). In the MT encoding module, an off‐resonance pulse is applied after excitation, prior to acquisition. If the offset frequency of the applied pulse is large relative to its peak amplitude and at the same time permits any direct saturation to be neglected, this enables partial saturation of the broad absorption lineshape of the macromolecule with minimum saturation of the liquid pool and concurrently satisfies the condition required for the Bloch‐Siegert shift,^20^ which we define as the BTS (Bloch‐Siegert and magnetization Transfer Simultaneously) criteria.^15^ Accordingly, the partially saturated macromolecule results in a decrease in the observable transverse magnetization of the free pool (Figure 2B). The spin evolution module of the acquisition sequence consists of a multi‐echo acquisition scheme. Due to a combination of spin–spin relaxation and field inhomogeneity, signal magnitude decays as a function of TE (${\mathrm{TE}}_{n}$). Employing an exponential decay model, one can evaluate the time evolution of the signal magnitude to quantify $T_{2}^{*}$.

Referring the reader to^15^ for detailed derivations, decoupling the excitation, saturation, relaxation and magnetization exchange process within repetition interval TR and arranging them into a sequence of events,^21^ invoking steady‐state, one can derive the magnitude term of the analytical signal model:

(6) $$\left|S\left({\mathrm{TE}}_{n}\right)\right|=\rho M_{0}\left(1-f\right)\sin\alpha \frac{1-\left(E_{1}^{F}A+E_{1}^{R}B\right)}{1-\left(E_{1}^{F}A\cos\alpha +E_{1}^{R}E_{W0}E_{W}C\right)}e^{-\frac{{\mathrm{TE}}_{n}}{T_{2}^{*}}}$$

where

$$A=1-f+fE_{k}-E_{k}E_{1}^{R}E_{W}E_{W0}$$

$$B=f-fE_{k}+E_{k}E_{W}E_{W0}$$

$$C=f+E_{k}-fE_{k}$$

$$E_{1}^{F\left[R\right]}=e^{-\frac{\mathrm{TR}}{T_{1}^{F\left[R\right]}}}$$

$$E_{W0}=e^{-\left\langle W\left(0\right)\right\rangle \tau_{\mathrm{exc}}}$$

$$E_{W}=e^{-\left\langle W\left(\Delta_{\mathrm{off}}\right)\right\rangle \tau_{\mathrm{BTS}}}$$

(7) $$E_{k}=e^{-\frac{1}{1-f}k_{R}\mathrm{TR}}=e^{-\frac{1}{f}k_{F}\mathrm{TR}}=e^{-\mathrm{kTR}}$$

where ρ is a scaling term, α is the excitation flip angle (FA) arising from an excitation pulse of length $\tau_{\mathrm{exc}}$, f is the macromolecular proton fraction with respect to the total magnetization $M_{0}$ ($=M_{0}^{F}+M_{0}^{R}$) and $\tau_{\mathrm{BTS}}$ is the BTS inducing pulse width applied at offset frequency $\Delta_{\mathrm{off}}$.

### 2.3 Signal phase modeling

The phase of the free pool transverse magnetization linearly increases commensurate with the amount of static field deviation it is exposed to. Factors that can contribute to this deviation include inhomogeneity of the main field, chemical shift and tissue susceptibility. Magnetic susceptibility (χ) is a tissue specific property which generates an additional magnetic dipole moment in response to an externally applied magnetic field. In the presence of $B_{0}\hat{z}$, this induces a field variation due to the contribution of the surrounding tissue susceptibility sources (Figure 1B). Considering only isotropic magnetic susceptibility sources,^22^, ^23^ for a free pool spin located at $\overset{⃑}{r}$, the z‐component of this field variation is given by^24^

(8) $$\Delta B_{\chi }\left(\vec{r}\right)=\frac{\mu_{o}}{4\pi }\int_{V^{\prime }}\left(3\frac{m_{z}\left(\vec{r}\right){\left(z-z^{\prime }\right)}^{2}}{{\left|\vec{r}-{\vec{r}}^{\prime }\right|}^{5}}-\frac{m_{z}\left(\vec{r}\right)}{{\left|\vec{r}-{\vec{r}}^{\prime }\right|}^{3}}\right)d^{3}{\vec{r}}^{\prime }$$

where ${\vec{r}}^{\prime }$ denotes source location of a magnetic dipole moment, $m_{z}\left(\vec{r}\right)\approx \chi \left(\vec{r}\right)\frac{B_{0}}{\mu_{o}}$ is the induced magnetic dipole moment per unit volume at $\vec{r}$ assuming $\chi \left(\vec{r}\right)\ll 1$ and $\mu_{o}$ is the magnetic permeability of free space. Eq. (8) can be rewritten as a convolution between $m_{z}\left(\vec{r}\right)$ and the dipole kernel $d\left(\vec{r}\right)=\frac{1}{4\pi }\frac{3\cos^{2}\theta -1}{{\left|\vec{r}-{\vec{r}}^{\prime }\right|}^{3}}$ ^25^

(9) $$\Delta B_{\chi }\left(\vec{r}\right)=\mu_{o}m_{z}\left(\vec{r}\right)*d\left(\vec{r}\right)$$

where θ is the angle between $\vec{r}-{\vec{r}}^{\prime }$ and $\vec{z}$. Utilizing the convolution theorem, Eq. (9) becomes

(10) $$\Delta B_{\chi }\left(\vec{r}\right)=B_{0}{ℱ}^{-1}\left\{X\left(\vec{k}\right)D\left(\vec{k}\right)\right\}$$

where $X\left(\vec{k}\right)$ and $D\left(\vec{k}\right)=\left\{\begin{array}{l} \frac{1}{3}-\frac{k_{z}^{2}}{{\left|\vec{k}\right|}^{2}}\\ 0 \end{array}\right.\begin{array}{c} \text{for}\;\vec{k}\neq 0\;\\ \text{for}\;\vec{k}=0 \end{array}$ are the Fourier transforms of $\chi \left(\vec{r}\right)$ and $d\left(\vec{r}\right)$, respectively, and ${ℱ}^{-1}$ the inverse Fourier transform operator.

Assuming negligible chemical shift and microstructure phase,^26^ and taking into account the $B_{1}^{+}$ proportional phase obtained from the BTS pulse, the phase term of the signal model is given by

(11) $$e^{i∠S\left({\mathrm{TE}}_{n}\right)}=e^{i\varphi_{\mathrm{BS}}}e^{i\varphi_{{\mathrm{TE}}_{n}}}$$

where

$$\varphi_{\mathrm{BS}}=B_{1,\text{peak}}^{2}\int_{0}^{\tau_{\mathrm{BTS}}}\frac{{\left(\gamma B_{1,\text{normalized}}\left(t\right)\right)}^{2}}{2\left(2\pi \Delta_{\mathrm{off}}\right)}\mathrm{dt}=B_{1,\text{peak}}^{2}K_{\mathrm{BS}}$$

(12) $$\varphi_{{\mathrm{TE}}_{n}}=\varphi_{o}+\gamma \Delta B_{\mathrm{bg}}{\mathrm{TE}}_{n}+\gamma \Delta B_{\chi }{\mathrm{TE}}_{n}$$

$B_{1,\text{peak}}$ and $B_{1,\text{normalized}}\left(t\right)$ are the peak amplitude and amplitude modulation function normalized to 1, respectively, of the BTS pulse. $\varphi_{\mathrm{BS}}$ is the phase induced by the Bloch‐Siegert shift that can be used to estimate $B_{1}^{+}$ field inhomogeneity. $\varphi_{{\mathrm{TE}}_{n}}$ is a ${\mathrm{TE}}_{n}$‐dependent phase consisting of a TE‐independent transceiver component ($\varphi_{o}$) and TE‐dependent components originating from background field inhomogeneity ($\Delta B_{\mathrm{bg}}$) and susceptibility ($\Delta B_{\chi }$). The ${\mathrm{TE}}_{n}$‐dependent phase component can be applied to tissue susceptibility models for quantification.

## 3 METHODS

### 3.1 Signal magnitude processing pipeline

**FIGURE 3:**
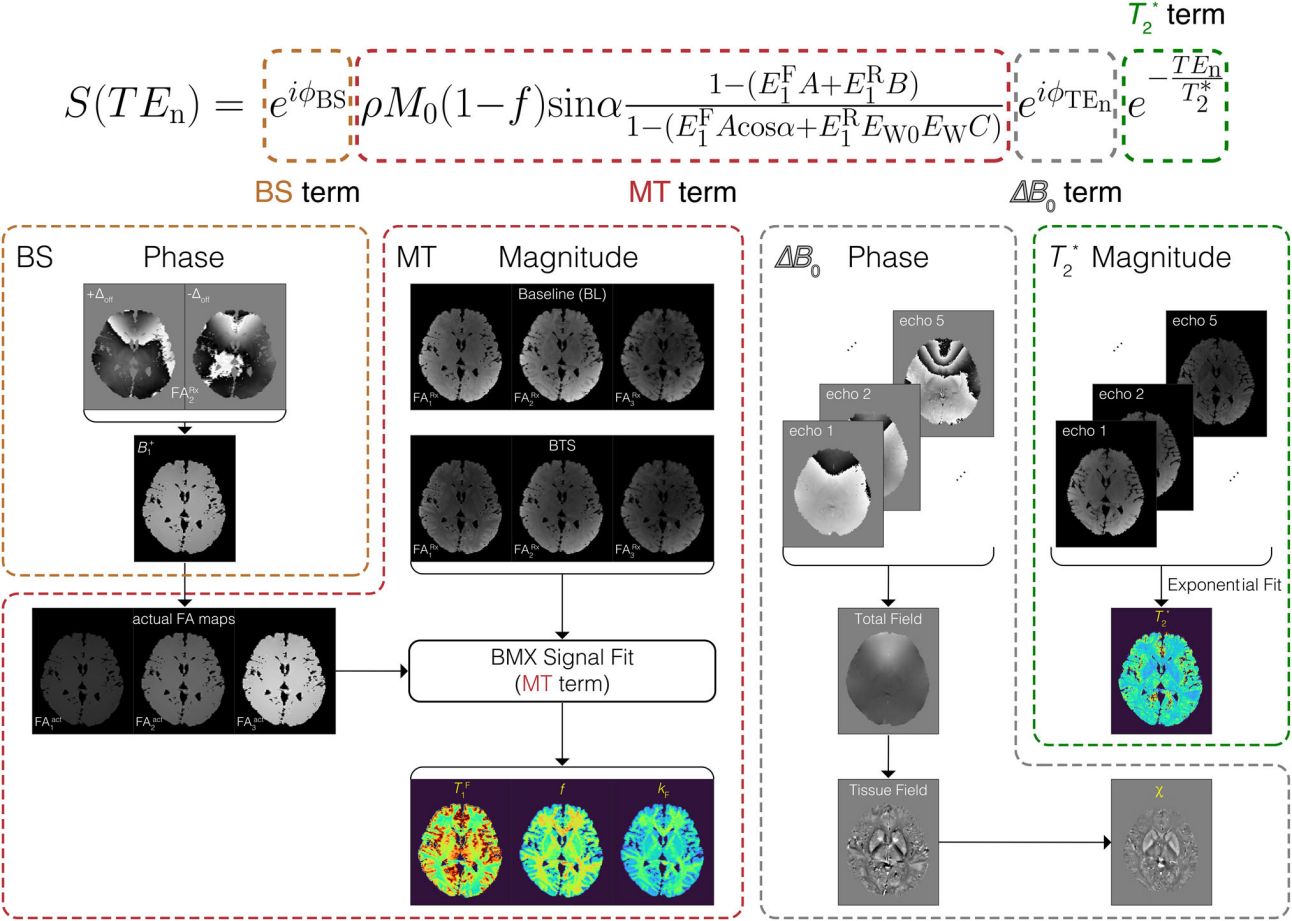
The signal model, composed of Bloch‐Siegert (BS), magnetization transfer (MT), $\Delta B_{0}$ and $T_{2}^{*}$ terms, is fully utilized to quantify MT parameters, tissue susceptibility and $T_{2}^{*}$. Acquisitions with off‐resonance BTS pulse applied at multiple excitation prescription flip angles (${\mathrm{FA}}_{n}^{\mathrm{Rx}}$) are obtained. An additional BTS acquisition at prescription angle closest to the Ernst angle (${\mathrm{FA}}_{2}^{\mathrm{Rx}}$ in this example) is used to remove any $B_{0}$ inhomogeneity and chemical shift dependence on the BS shift. The resulting phase image, corresponding to the BS term, is used to derive a $B_{1}^{+}$ map, which is then utilized to generate actual FA maps that reflect $B_{1}^{+}$ inhomogeneity. The spatially varying actual FA maps, BTS magnitude images and separately acquired baseline images (BTS pulse not applied at multiple excitation prescription angles) are used to fit the MT term pixel by pixel, generating $T_{1}^{F}$, f and 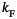 maps. The ${\mathrm{TE}}_{n}$‐dependent $\Delta B_{0}$ phase term is used estimate total field, which is then applied to remove background field. The estimated tissue field is subsequently inverted to estimate tissue susceptibility (χ). Mono‐exponential decay is used to fit ${\mathrm{TE}}_{n}$‐dependent $T_{2}^{*}$ magnitude term to extract effective $T_{2}$.

The magnitude term processing pipeline is used to quantify magnetization transfer effects. Referring to Figure 3, two sets of data, one without (baseline [BL]) and one with BTS applied, each at multiple flip angles, are acquired. Spatially varying actual FA maps, obtained through the phase processing pipeline described below, are combined with both the BL and BTS magnitude images and utilized to pixel‐wise fit the magnitude term of the signal model that embodies MT (Figure 3, **MT term**) to estimate MT parameter maps $T_{1}^{F}$, f and $k_{F\left[R\right]}$ assuming $T_{1}^{R}$ = 1 s, $T_{2}^{R}$ = 12 μs.^9^, ^10^, ^21^, ^27^ To calculate the average saturation rate of the macromolecule pool, the super‐Lorentzian absorption line shape given by:

(13) $$G_{\mathrm{SL}}\left(\Delta \right)=\int_{0}^{1}\sqrt{\frac{2}{\pi }}\frac{T_{2}^{R}}{\left|3u^{2}-1\right|}e^{-2{\left[\frac{2\pi \Delta T_{2}^{R}}{3u^{2}-1}\right]}^{2}}\mathrm{du}$$

where the on‐resonance singularity of the above equation was approximated as 1.4 × 10^−5^ s^−1^ ^28^. $T_{2}^{*}$ maps were additionally obtained by fitting the multi‐echo magnitude data to a mono‐exponential decay model (Figure 3, $T_{2}^{*}$ **term**) using linear regression.

### 3.2 Signal phase processing pipeline

The phase data obtained from the multi‐echo acquisitions are applied to the phase evolution term (Figure 3, $∆B_{0}$ **term**) to extract tissue magnetic susceptibility maps. In this work, ROMEO^29^ was used to estimate total field, followed by generation of a brain mask^30^ which was subsequently used to remove the background field contribution to isolate the susceptibility induced field based on VSHARP.^31^ This remaining field was then inverted using MEDI^32^ to generate tissue susceptibility maps.^33^, ^34^

The Bloch‐Siegert shift term (Figure 3, **BS term**) is used to estimate $B_{1}^{+}$, where an additional acquisition with the BTS pulse offset frequency symmetrically applied about the carrier frequency at a flip angle closest to the Ernst angle is performed to eliminate phase accumulation not originating from the Bloch‐Siegert phenomena.^16^ From this, a $B_{1}^{+}$ map is extracted from the phase image which are then combined with the prescribed FA to generated actual FA maps used in the above magnitude processing pipeline for MT quantification.

### 3.3 Experiments

In vivo study of the human brain was carried out on five healthy volunteers, ages 34–38 years, to test the feasibility of our method under a protocol approved by our institution's institutional review board. Experiments were carried out on a 3T clinical scanner (Skyra, software version XA30, Siemens Healthineers, Erlangen, Germany) equipped with a 32‐channel head receiver. Common 3D sequence parameters used in both BL and BTS acquisitions were: matrix size = 192 × 84 × 72 yielding 1.1 × 2.2 × 2.2 mm resolution along the axial direction, TE/TR = 12/40 ms and prescribed excitation flip angles 10°, 20°, 40°. 10 dummy scans and a reverse centric *k*‐space acquisition scheme were carried out to ensure contrast was measured in steady‐state. For BTS acquisitions, a fermi pulse ($B_{1}\left(t\right)=\frac{B_{1,\text{peak}}e^{i\Delta_{\mathrm{off}}t}}{1+\exp\left\{\frac{\left|t-\tau_{\mathrm{BTS}}/2\right|-0.00276875}{0.00017}\right\}}$)^35^ 8 ms in length with $B_{1,\text{peak}}$ = 7.3 μT ($B_{1,\mathrm{rms}}$ = 5.93 μT) and carrier offset 4 kHz was used. In the BL acquisition nearest the Ernst angle (20°), 5 TEs spanning 6–30 ms with 6 ms echo spacing were acquired using a monopolar scheme with matrix size doubled in both phase and slice encode directions yielding 1.1 × 1.1 × 1.1 mm resolution for tissue susceptibility mapping.^34^ For all acquisitions, parallel imaging was applied along the slice encode direction using acceleration factor 2 with 24 calibration lines, resulting in a total scan time of 28 min.

### 3.4 Regional analysis

Regional analysis was performed using segmentation software^36^ to identify white matter (WM) and gray matter (GM) regions. Representative WM and GM region of interest (ROI) regions that are commonly assessed collectively in both qMT and QSM were chosen in consideration of validating our method. In these regions, the mean and SD of MT parameters $T_{1}^{F}$, f, $k_{F}$, tissue susceptibility χ and $T_{2}^{*}$ were evaluated.

## 4 RESULTS

**FIGURE 4:**
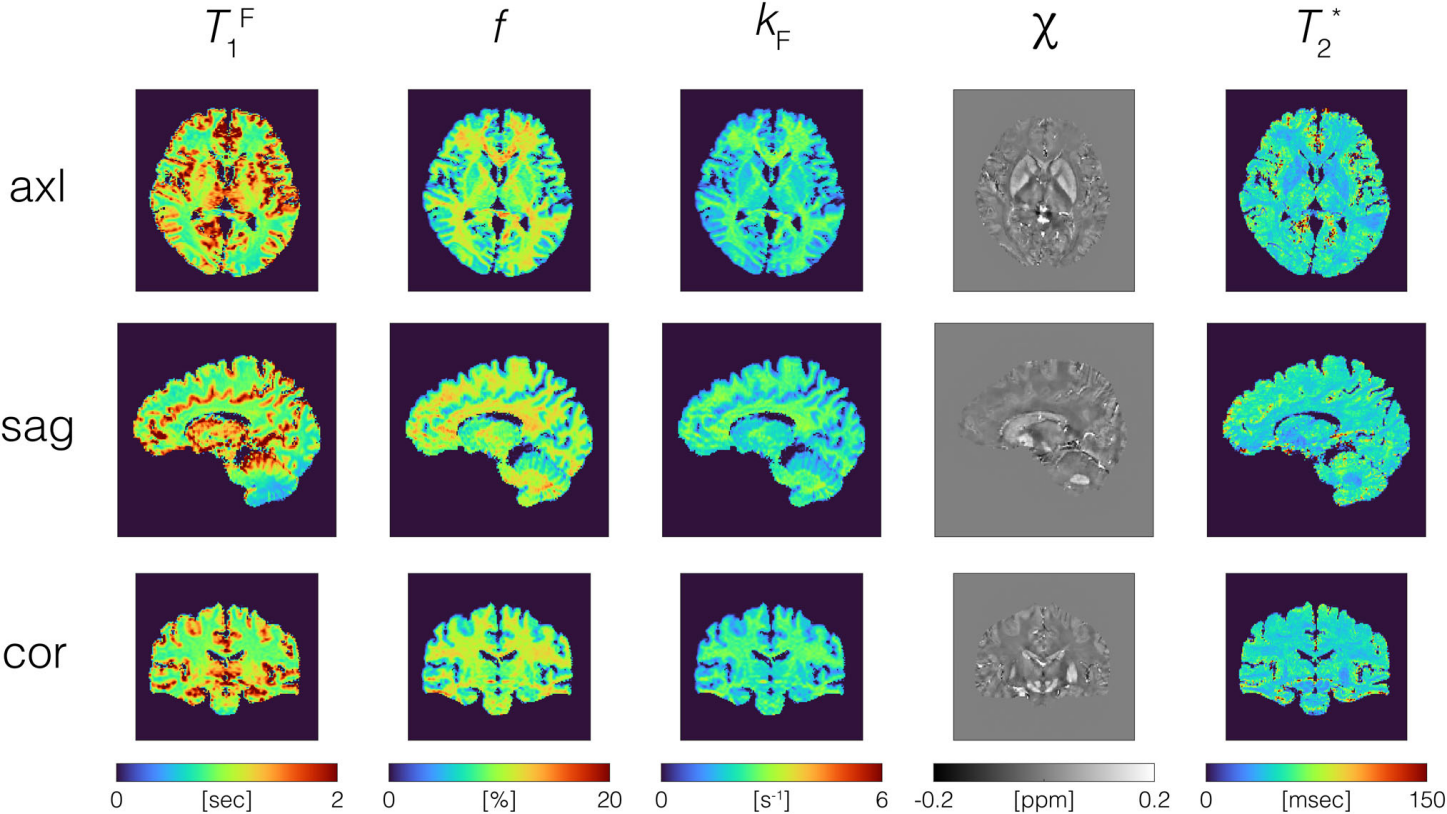
Estimated binary spin‐bath parameters, tissue susceptibility and $T_{2}^{*}$ maps taken along the axial (first row), sagittal (second row) and coronal (third row) view from an in vivo study. Corresponding $T_{1}^{F}$ (column 1), macromolecular fraction (column 2), rate constant (column 3), tissue susceptibility (column 4) and $T_{2}^{*}$ (column 5) obtained from our method. White matter (WM) regions show greater macromolecule content compared to gray matter (GM) regions. $T_{2}^{*}$ values were larger in GM regions compared to WM regions. Presence of diamagnetic lipids in myelin WM regions contributes to negative tissue susceptibility, while paramagnetic iron contributes to positive tissue susceptibility in GM regions.

Examples of in vivo MT parameters, susceptibility and $T_{2}^{*}$ maps of the brain are presented in Figure 4. Referring to the regional analysis results shown in Table 1, compared to GM regions, myelin rich WM exhibit lower $T_{1}^{F}$ values accompanied by higher macromolecular proton fractions and consequently higher transfer rates from F to R. The presence of diamagnetic lipids in myelin WM regions contributes to negative tissue susceptibility, while paramagnetic iron contributes to positive tissue susceptibility in GM regions.^37^ Overall $T_{2}^{*}$ values were larger in GM regions compared to WM regions. Comparing our results in different regions of the brain with those reported in previous literature^15^, ^31^, ^38^, ^39^ show good agreement. Note that the reference methods obtained either MT or tissue susceptibility parameters separately.

**TABLE 1:** Data acquired are presented as mean ± SD.

Region‐of‐interest	$T_{1}^{F}$ [s]	f [%]	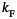 [s^−1^]	*χ* [ppm]	$T_{2}^{*}$ [ms]
White matter region					
Corpus callosum, genu	0.814 ± 0.170 (0.969 ± 0.219)^a^	11.46 ± 1.84 (13.15 ± 3.56)^a^	2.485 ± 0.28 (3.093 ± 0.86)^a^	−0.0123 ± 0.009 (−0.033 ± 0.013)^b^	42 ± 3.21 (N/A)
Corpus callosum, splenium	0.988 ± 0.269 (1.020 ± 0.255)^a^	11.73 ± 1.76 (12.23 ± 3.30)^a^	2.519 ± 0.20 (2.884 ± 0.80)^a^	−0.0298 ± 0.0037 (−0.038 ± 0.013)^b^	41 ± 1.83 (41.49)^c^
Frontal white matter	0.868 ± 0.170 (0.864 ± 0.179)^a^	10.89 ± 2.38 (13.22 ± 2.11)^a^	2.353 ± 0.19 (3.100 ± 0.51)^a^	−0.0042 ± 0.0017 (N/A)	48 ± 1.49 (53.79 ± 4.66)^d^
Gray matter region					
Caudate nucleus	1.305 ± 0.206 (1.317 ± 0.184)^a^	6.99 ± 1.21 (6.69 ± 1.24)^a^	1.51 ± 0.16 (1.550 ± 0.29)^a^	0.0339 ± 0.007 (0.019 ± 0.012)^b^	53 ± 3.57 (40.45 ± 4.5)^d^
Putamen	1.227 ± 0.256 (N/A)	7.74 ± 1.27 (N/A)	1.67 ± 0.20 (N/A)	0.0242 ± 0.0043 (0.043 ± 0.020)^b^	48 ± 5.19 (40.70 ± 3.67)^d^
Cerebral cortex	1.211 ± 0.270 (1.408 ± 0.178)^a^	6.28 ± 2.20 (6.50 ± 2.16)^a^	1.352 ± 0.23 (1.506 ± 0.51)^a^	0.0034 ± 0.0019 (N/A)	63 ± 2.80 (N/A)

^a^ Values reported from Jang et al.,^15^ refer to reference for method details.

^b^ Values reported from Li et al.,^31^ refer to reference for method details.

^c^ Values reported from Sati et al.,^37^ refer to reference for method details.

^d^ Values reported from Peran et al.,^38^ refer to reference for method details.

## 5 DISCUSSION AND CONCLUSION

We have introduced a new unified acquisition and modeling strategy that fully leverages the magnitude and phase of the acquired MR signals from a spoiled gradient echo‐scheme for multiparametric qMRI. Combining the binary spin‐bath MT model and tissue susceptibility model, we have applied and demonstrated its capability to simultaneously quantify magnetization transfer and tissue susceptibility with $B_{1}^{+}$ field correction through the Bloch‐Siegert phenomena in a unified framework. This method has been successfully demonstrated to image whole brain 3D MT and susceptibility at a reasonable scan time and the values obtained in this study agree with previously reported literature values.

Our new method provides a multiparametric approach to simultaneously deduce tissue parameters for both MT and tissue susceptibility. In contrast to our original BTS method, which focused on MT quantification, this new method allows for additional assessment of tissue susceptibility and $T_{2}^{*}$ through a multi‐echo scheme in the spoiled gradient‐echo‐based BTS sequence. More specifically, the BTS sequence requires a relatively long TR due to three facts: (1) enabling the necessary high signal‐to‐noise level, (2) minimizing the influence of incomplete spoiling on $T_{1}^{F}$ estimation using a large spoiler gradient and (3) facilitating high specific absorption rate (SAR) due to the MT modulation pulse depositing high RF energy. Multi‐echo acquisition can be leveraged to take advantage of this idle time window to additionally encode spin evolution during the long TR with an increased duty cycle but no cost of additional acquisition time. With this new scheme of simultaneous MT and tissue susceptibility assessment, our approach offers new opportunities to specify tissue pathology in tissues. One potential application could be investigating multiple sclerosis in the brain and spinal cord. While individual MT and tissue susceptibility parameters have been found to provide interpretation of tissue damage, such as using MT for assessing demyelination^40^ and using susceptibility for assessing iron deposition in MS,^41^ a combined assessment of using both may provide better interpretation of disease progression and staging in MS. Nevertheless, this necessitates additional studies to validate our method's robustness to various pathological conditions such as MS, or across larger sample sizes and more diverse clinical populations. In addition, further optimization of our method such as greater acceleration and refining acquisition strategies and protocol to better suite our studies may be necessary for it to become clinically applicable within a realistic scan time for these additional studies. Extending our method to other non‐brain organs is also possible. However, other factors need to be further considered such as body motion and chemical shift induced information (e.g., fat) for modeling MT^42^ and tissue susceptibility.^43^

In this study, a super‐Lorentzian absorption line shape was used to calculate the average saturation rate of the macromolecule pool, which is well accepted in adequately characterizing the saturation of macromolecules in tissues such as WM and GM when applying the binary spin‐bath model.^44^ However, recent studies have shown that on‐resonance saturation and dipolar order effects contribute to biased qMT parameter estimates.^14^ Although on‐resonance saturation effects are explicitly modeled in our method, the on‐resonance singularity of the super‐Lorentzian function (Eq. 13) was estimated by extrapolating it from Δ = 1 kHz to the asymptotic limit Δ ➔ 0.^28^ The potential inaccuracy of the on‐resonance saturation and the use of single‐offset saturation could explain the reason of the discrepancy between our qMT parameter estimates and those reported in recent literature.^14^ Utilizing recent simulation‐based approaches^45^ to more accurately model on‐resonance saturation effects and applying simultaneous dual‐offset saturation to nullify dipolar order and associated relaxation effects are required to validate this hypothesis and will be investigated in the future.

One challenge of multiparametric approaches, including ours, is the long acquisition time, making it difficult to be clinically translatable. GRAPPA^46^ with two‐fold acceleration was applied in our method to accelerate image acquisition; however, careful consideration of the trade‐off between scan time reduction and quantitative accuracy must be taken given parallel imaging not only reduces overall SNR, but also exhibits spatial variance.^47^ This can lead to potential quantification errors in MT and tissue susceptibility maps. Several strategies can be further considered to improve scanning efficiency. For higher acceleration factors, utilizing better *k*‐space acquisition strategies such as CAIPIRHINIA,^48^ compressed‐sensing‐based incoherent undersampling,^49^ non‐Cartesian sampling^50^ or other improved spatial encoding methods^51^ might result in better noise performance. Model‐based reconstruction approaches can also be used and further combined with parallel imaging^45^ to decrease scan time. More recently, deep learning approaches^52^, ^53^, ^54^ have been introduced for accelerated qMRI with improved noise performance and quantification accuracy, and they are potential avenues worth further investigation for accelerating simultaneous MT and tissue susceptibility mapping.

We have introduced a new unified acquisition and modeling strategy that fully leverages the magnitude and phase of the acquired MR signals. Our method was applied to demonstrate simultaneous quantification of magnetization transfer, tissue susceptibility and $T_{2}^{*}$ of the brain tissue in this study, showing potential applications to improve tissue microstructure and microenvironment assessment.
